# VegNet: An organized dataset of cauliflower disease for a sustainable agro-based automation system

**DOI:** 10.1016/j.dib.2022.108422

**Published:** 2022-06-26

**Authors:** Umme Sara, Aditya Rajbongshi, Rashiduzzaman Shakil, Bonna Akter, Mohammad Shorif Uddin

**Affiliations:** a Department of Computer Science and Engineering, National Institute of Textile Engineering and Research, Dhaka, Bangladesh; b Department of Computer Science and Engineering, Daffodil International University, Dhaka, Bangladesh; c Department of Computer Science and Engineering, Jahangirnagar University, Dhaka, Bangladesh

**Keywords:** Computer vision system, Agriculture, Feature extraction, Machine learning

## Abstract

Cauliflower, a winter seasoned vegetable that originated in the Mediterranean region and arrived in Europe at the end of the 15th century, takes the lead in production among all vegetables. It's high in fiber and can keep us hydrated, and have medicinal properties like the chemical glucosinolates, which may help prevent cancer. If proper care is not given to the plants, several significant diseases can affect the plants, reducing production, quantity, and quality. Plant disease monitoring by hand is extremely tough because it demands a great deal of effort and time. Early detection of the diseases allows the agriculture sector to grow cauliflower more efficiently. In this scenario, an insightful and scientific dataset can be a lifesaver for researchers looking to analyze and observe different diseases in cauliflower development patterns. So, in this work, we present a well-organized and technically valuable dataset “VegNet’ to effectively recognize conditions in cauliflower plants and fruits. Healthy and disease-affected cauliflower head and leaves by black rot,downy mildew, and bacterial spot rot are included in our suggested dataset. The images were taken manually from December 20th to January 15th, when the flowers were fully blown, and most of the diseases were observed clearly. It is a well-organized dataset to develop and validate machine learning-based automated cauliflower disease detection algorithms. The dataset is hosted by the Institute – National Institute of Textile Engineering and Research (NITER),the Department of Computer Science and Engineering and is available at the link following: https://data.mendeley.com/datasets/t5sssfgn2v/3.

## Specifications Table

**Table:** 

Subject	Computer Science
Specific subject area	Image analysis and classification, feature extraction, feature ranking and computer vision
Type of data	images
How the data were acquired	Cauliflower-growing locations are primarily selected by where the production is high. We chose the location Manikganj (vegetable producing zone near by Dhaka) where various proprietors' vast acreage of acres is available to cultivate vegetables. From the end of December to the middle of January, we collected a large amount of images of disease-affected and healthy cauliflower and leaves. According to an expert opinion, finally we worked on the field with a Digital Camera to acquire raw images.
Data format	Raw JPG
Description of data collection	Previously, no image had ever been used in any experiment. All of the image samples were shot over the period of ten days in a variety of light and atmospheric conditions and then stored as a jpg format. We diligently captured all the images very meticulously at the peak of cauliflower maturity, according to the directions of a plant pathology expert from the Bangladesh Rice Research Institute (BARI) in Gazipur.
Data source location	**Location:** Singair**Zone:** Manikganj, Dhaka**Country:** Bangladesh
Data accessibility	**Repository name:** Mendeley Data**Data identification number (permanent identifier, i.e., DOI number):** 10.17632/t5sssfgn2v.3**Direct link to the dataset:**https://data.mendeley.com/datasets/t5sssfgn2v/3

## Value of the Data

• This comprehensive dataset ‘VegNet’ consists of 8000+ images and can identify the cauliflower fruit diseases with bare eyes. As a result, the researchers can contribute efficiently to the analysis of data and detect the diseases at early stages.
• Different machine and deep learning-based feature ranking algorithms can be used to classify,compare, test, and estimate data to diagnose the disease-affected cauliflower fruits.
• This dataset can be used to create high-quality cauliflower classification and detection applications.
• The dataset can be used to develop disease classification apps that will assist farmers incorrectly producing their crops, which will benefit the agriculture industry, wholesalers, customers, and vegetable export firms broadly.

## 1 Data Description

The vegetable Cauliflower is the world's second most popular 'cole' crop after cabbage, but it is the most popular in Bangladesh [1]. Cauliflower is a seasonal crop grown primarily by farmers on farmland. It is high in minerals such as iron, magnesium, phosphorus, potassium, sodium, and vitamins A and B1 [2]. Furthermore, cauliflower has a low-fat content compared to a high fiber, Vitamin B9, L-Ascorbic acid content, and water makes it a high nutritional density [3].

The diseases of the cauliflower plants are crucial in decreasing vegetable production and causing loss to the agro-economy. The cauliflower plant is affected by the diseases like bacterial spot rot, blackleg, black rot, clubroot, cauliflower mosaic virus, downy mildew, powdery mildew, black rot, sclerotinia stem rot, white rust, cauliflower mosaic, ringspot, etc. Furthermore, we observed that downy mildew, black rot, and bacterial spot rot, among other diseases, frequently harm cauliflower and leaves. [4]. In this case, our proposed dataset can be a state-of-art reference for developing algorithms to the early recognition of different cauliflower diseases in agriculture. This article includes a large dataset with 656 original images and 7360 augmented images of healthy and disease-affected cauliflower and leaves. The image distribution of the dataset is specified in Table 1.

**Table 1:** Class wise dataset distribution.

Class Name	Number of Original Images	Number of Augmented Images
Downy Mildew	177	1770
Black Rot	100	1800
Bacterial Spot Rot	173	1730
Disease free	206	2060
Total	656	7360

### 1.1 Diseases Details

#### 1.1.1 Downy Mildew

**Fig. 1:**
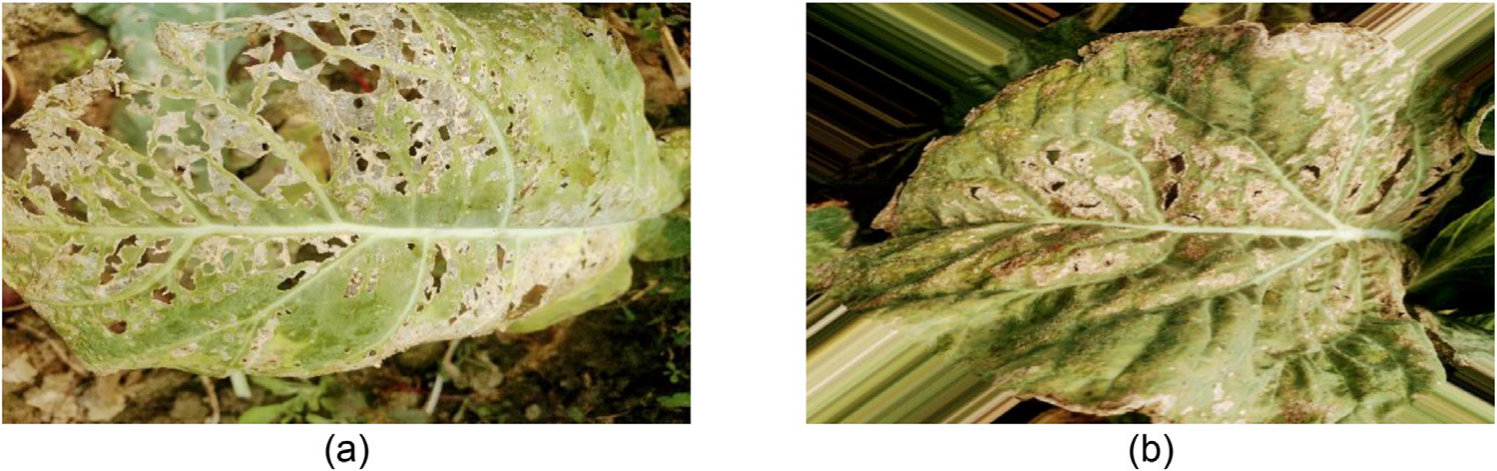
Visualization of (a) original and (b) augmented images of Downy Mildew affected cauliflower leaves.

*Peronospora parasitica*, an oomycete, is the principal cause of downy mildew [5]. It causes white, yellow, or brownish patches on the top surfaces of older leaves, as well as downy grey mold on the undersides, by penetrating the vegetation through lesions and intrinsic holes (this eventually releases more spores). As these areas deepen in color, the leaf dies (Fig. 4(d)). Moisture and low temperatures are required for downy mildew to grow. Fig. 1 shows the images of downy mildew disease-affected cauliflower leaves.

#### 1.1.2 Black Rot

**Fig. 2:**
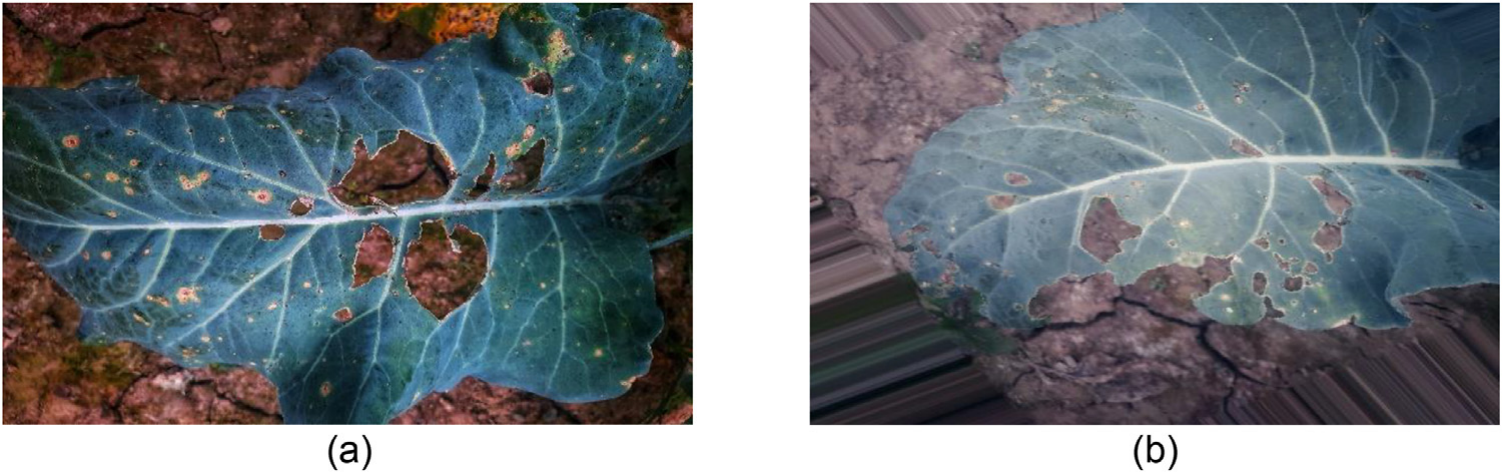
Visualization of (a) original and (b) augmented images of Black Rot disease affected cauliflower leaves.

Black rot is a bacterial disease that affects cauliflower plants worldwide, rendering them unfit for sale or consumption. After cruciferous vegetables begin to grow, symptoms of black rot may not appear for up to a month. Irregular, dull yellow spots form on the edges of leaves as the first sign of the disease. These blotches grow into V-shaped patches as the disease spreads, with the wide section of the "V" at the leaf's border and the leaf's attachment point to the plant. Fig. 2 presents the original images of black rot-affected cauliflower leaves.

#### 1.1.3 Bacterial Spot Rot

**Fig. 3:**
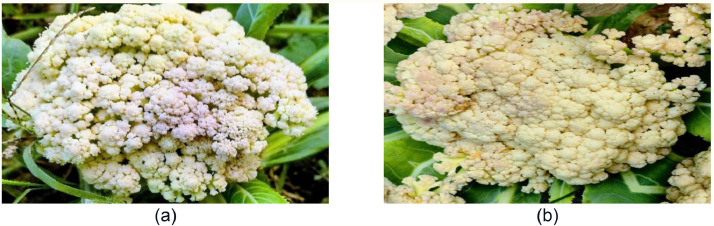
Visualization of (a) original and (b) augmented images of Bacterial Spot Rot affected cauliflower.

Lesions on flower heads that have been drenched in water grow to form a large rotting mass; the surface of lesions frequently splits and releases a slimy substance that turns tan, dark brown, or black when exposed to air. Bacteria are easily transmitted on tools and irrigation water, and disease onset is favored by warm, moist circumstances. Images of bacterial spot rot-affected disease on cauliflower heads are shown in Fig. 3. As there are no chemical treatments for bacterial soft rot, control is reliant on agricultural practices: rotate crops, grow in well-draining soils or raised beds, pick heads only when they are dry, and avoid negative charges during harvest.

#### 1.1.4 Disease-free Cauliflower

**Fig. 4:**
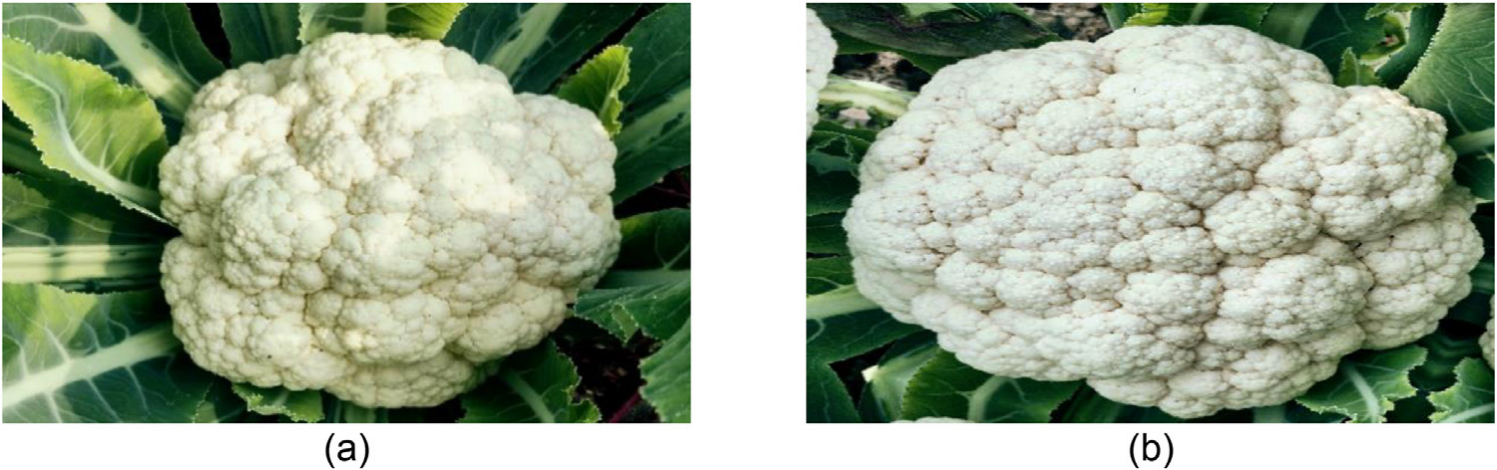
. Visualization of (a) original and (b) augmented images of fresh cauliflower.

The white head at the end of the cauliflower plant is the fruit. Fluorescence is an immature cluster of flowers that creates a firm, succulent "curd," or head, at the end of the terminal, reaches about 0.5 meters (1.5 feet) in height and bears large circular leaves that look like collards. Before harvest, the broad leaves are sometimes strung together to shade the curd and prevent discoloration. It's the best time to collect fresh and disease-free cauliflowers while the head is complete before it starts to separate. Original images of fresh Cauliflower are presented in Fig. 4.

## 2 Materials, Procedures and Experimental Design

### 2.1 Image Capturing Device Specification

All images are captured with the assistance of a digital camera device, and the complete specifications are presented in Table 2.

**Table 2:** Camera Specification details.

Sl No.	Camera Particles	Details
1	Manufacturer	Sony
2	Model	Sony Cyber-Shot W-530
3	Pixel Effectivity	14 MegaPixels
4	Sensor in size	1/2.3 inch
5	Type of Sensor	Charge Coupled Device (CCD)
6	Focal Length	26–104 mm
7	ISO	Auto, 80, 100, 200, 400, 800, 1600, 3200
8	Max aperture	F2.7–5.7
9	Format	Motion JPEG
10	Max shutter speed	1/1600 sec

### 2.2 Acquired Image Features

We manually collected the photos from the farm field, making the dataset more real. Table 3 describes some of the image feature characteristics.

**Table 3:** Cauliflower image feature details.

Sl. No.	Features	Description
1.	Type	Vegetable
2.	Name	Cauliflower
3.	Color	White color cauliflower
4.	Collected area	Singair, Manikgonj
5.	Field Type	Square shape Plane land
6.	Area Size	5 acres of land
7.	Collection Process	Manually Captured by a Digital Camera
8.	Collection Time	20^th^ December, 2021–15^th^ January, 2022
9.	Image acquiring duration	10 days
10.	Image resolution	500 × 500 pixels

### 2.3 Image Acquisition Process

**Fig. 5:**
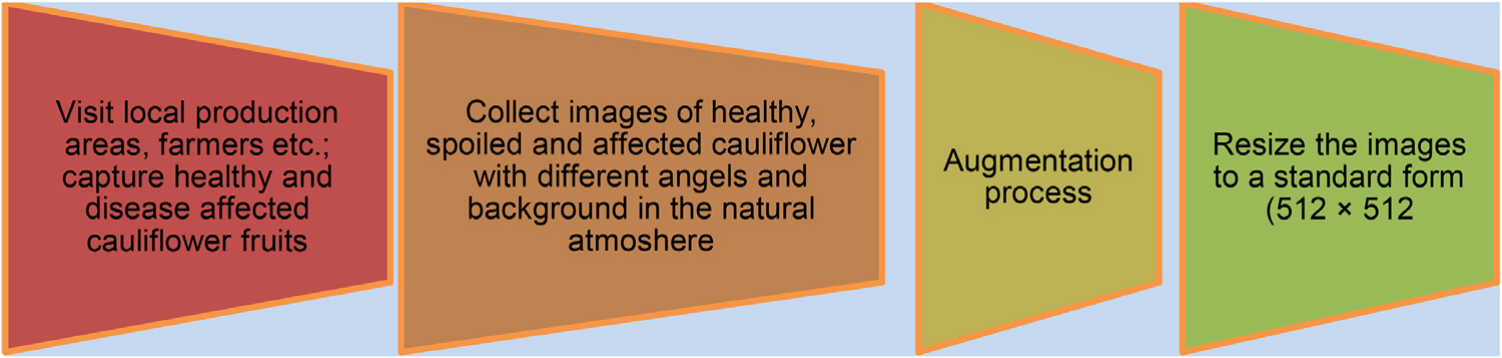
Cauliflower data acquisition process.

The image data acquisition process is shown in Fig. 5. The vegetable images were acquired using a digital camera with good quality resolution. We selected the local production areas and farmers at different locations. Finally, we chose the area Singair, Manikgonj and captured random clicked images in a jpeg format for 10 days. Lastly, the images were classified and stored for preprocessing. The images are captured in the natural atmosphere with different angles and backgrounds from December 2021 to January 2022. Images have been preprocessed using python script. In the preprocessing step, we changed the dimensions to 512 × 512, as the standard resolution required to build an object classification or object detection model. Besides, the Keras ImageDataGenerator technique has been applied to augment the original dataset as this method is mainly performed well for real-time images in the recent era. During the augmentation process, different parameters such as rotation, height_shift_range and shear_range, width_shift_range and shear_range, etc are tuned.

### 2.4 Experimental Procedure

**Fig. 6:**
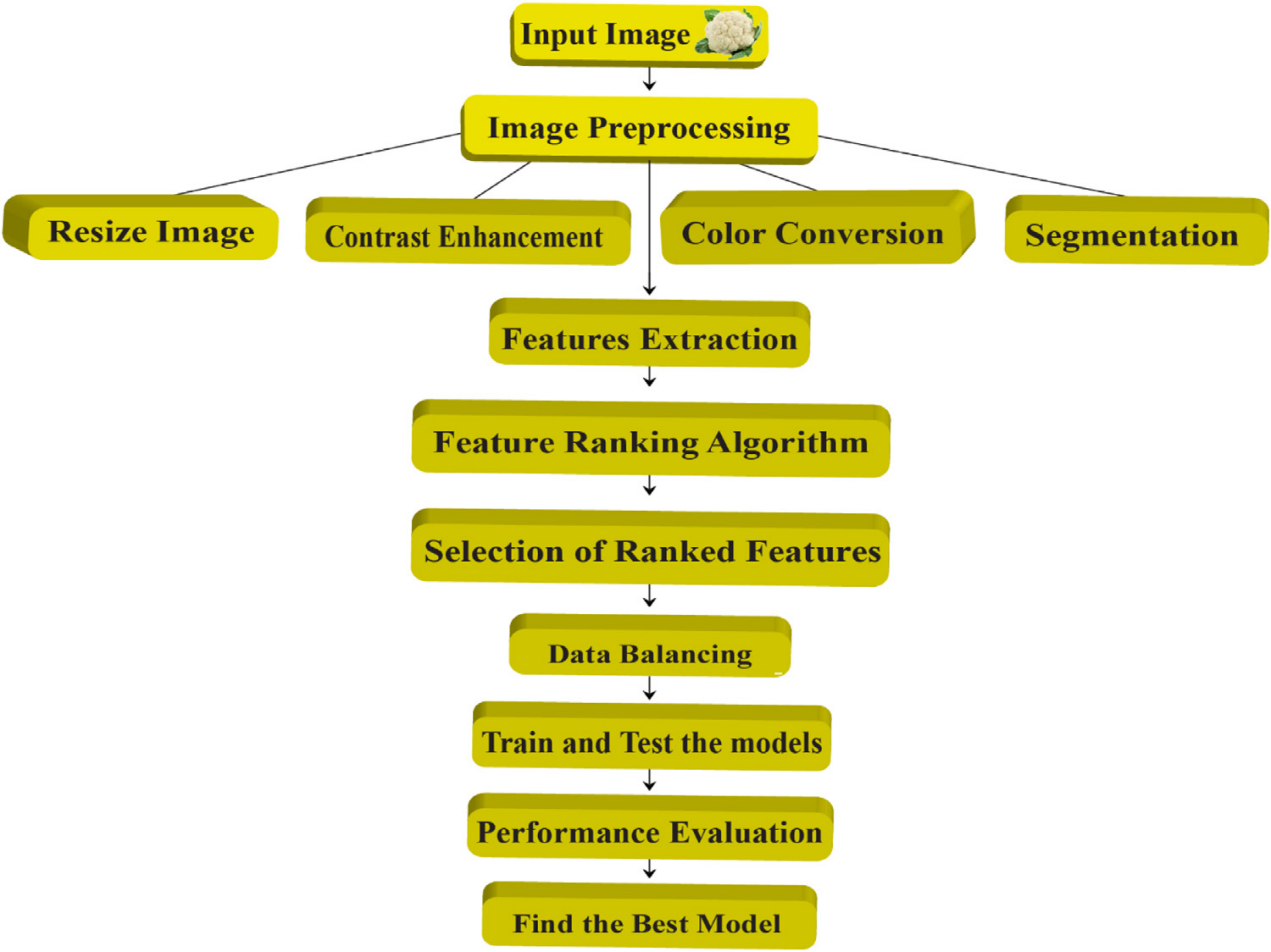
Feature ranking-based model generation process to detect cauliflower diseases.

We propose a feature ranking-based classical machine learning model to successfully exploit the dataset to create a state-of-the-art automation system. This worthwhile approach is now depicted in Fig. 6 and contains the following crucial steps:

1. Image preprocessing (resizing, contrast enhancement, color conversion, and segmentation).
2. Features extraction.
3. Apply the feature ranking method.
4. Top-ranked feature set selection.
5. Data balancing.
6. Model development and
7. Performance evaluation

As a result of the random clicks, we should now arrange the acquired images into uniform sizes and shapes. Adjust brightness, contrast, saturation, and hue, among other things, is what color improvement is all about. We need to shrink the images to 512 × 512 pixels using the bicubic interpolation approach to make the images consistent. The contrast of the resized images must then be improved. Instead, the color conversion step is carried out before segmentation. The various vital characteristics are retrieved when the segmentation processes are completed. Because all the features have a minor impact on categorization, feature ranking techniques are critical. Furthermore, different sets of ranked features can be constructed using feature ranking algorithms that will be used to generate the applicable model individually.

This dataset includes a variety of category classifications for early identification of three of the most commonly encountered diseases, with a variety of real-world applications. Furthermore, Fig. 6 depicts a typical flow procedure for identifying cauliflower disorders. Our future goal is to develop a disease identification model and validate its performance.

## Ethics Statements

There are no animal studies by any of the authors in this article.

## Ethical Approval (involvement of human subjects)

None of the authors in this article have conducted any human participant studies. The datasets used in this work are publicly available. Proper citation requirements should be followed when using these datasets.

## CRediT Author Statement

**Umme Sara:** Conceptualization –original draft preparation, Data curation, and Visualization; **Aditya Rajbongshi:** Methodology, Software; **Rashiduzzaman Shakil:** Validation; **BonnaAkter:** Writing and Investigation; **Mohammad Shorif Uddin:** Supervision, Reviewing, and Editing.

## Declaration of Competing Interest

The authors state that they have no competing financial or personal interests that could have influenced the work reported in this study.

## Data Availability

VegNet: An extensive dataset of cauliflower images to recognize the diseases using machine learning and deep learning models (Original data) (Mendeley Data).
